# On the Performance of Multiple Imputation Based on Chained Equations in Tackling Missing Data of the African α^3.7^-Globin Deletion in a Malaria Association Study

**DOI:** 10.1111/ahg.12065

**Published:** 2014-06-18

**Authors:** Nuno Sepúlveda, Alphaxard Manjurano, Chris Drakeley, Taane G Clark

**Affiliations:** 1 London School of Hygiene and Tropical MedicineLondon, UK; 2 Centre of Statistics and Applications of University of LisbonLisbon, Portugal; 3 National Institute for Medical ResearchMwanza Centre, Tanzania; 4 Joint Malaria ProgrammeMoshi, Tanzania

**Keywords:** Genotype imputation, multiple imputation based on chained equations, *HBA2* gene, malaria positivity

## Abstract

Multiple imputation based on chained equations (MICE) is an alternative missing genotype method that can use genetic and nongenetic auxiliary data to inform the imputation process. Previously, MICE was successfully tested on strongly linked genetic data. We have now tested it on data of the *HBA2* gene which, by the experimental design used in a malaria association study in Tanzania, shows a high missing data percentage and is weakly linked with the remaining genetic markers in the data set. We constructed different imputation models and studied their performance under different missing data conditions. Overall, MICE failed to accurately predict the true genotypes. However, using the best imputation model for the data, we obtained unbiased estimates for the genetic effects, and association signals of the *HBA2* gene on malaria positivity. When the whole data set was analyzed with the same imputation model, the association signal increased from 0.80 to 2.70 before and after imputation, respectively. Conversely, postimputation estimates for the genetic effects remained the same in relation to the complete case analysis but showed increased precision. We argue that these postimputation estimates are reasonably unbiased, as a result of a good study design based on matching key socio-environmental factors.

## Introduction

Missing genotypes are common in genetic association studies but often discarded from the analysis. This popular practice typically decreases statistical efficiency and power in comparison to an analysis where missing data are taken into account conveniently. It may also introduce estimation bias, particularly when the missing data pattern is not completely random. Since the advent of the HapMap project and the decrease in costs of genome-wide association studies (GWAS), several imputation methods have been proposed to deal with missing genotypes of typed or untyped single-nucleotide polymorphisms (SNPs; reviewed in Marchini & Howie, 2010). These imputation approaches aim to replace missing genotypes by plausible guesses, borrowing information not only from individuals with observed genotypes, but also from the underlying linkage disequilibrium (LD) and/or haplotype structure between genetic markers. To improve the quality of genotype prediction, one can also include available maps of recombination or reference data from HapMap in the imputation process. Three popular imputation software packages are BEAGLE (Browning, 2006; Browning & Browning, 2009), IMPUTE (Marchini et al., 2007; Howie et al., 2009), and MACH (Li et al., 2010), and their performance has been compared to each other in different data settings (Pei et al., 2008; Huang et al., 2009; Nothnagel et al., 2009; Chanda et al., 2012; Hancock et al., 2012). Notwithstanding the good performance of current genotype imputation methods in GWAS, they should be used with caution in alternative settings where the population under study is not so closely related to the ones in the HapMap database, as demonstrated by Jallow et al. (2009). The same applies to studies when there are weak to moderate LD patterns between the genetic markers with and without missing data, due to either limitations in the study design or the presence of latent population structures. In these situations, it seems more reasonable and sensible to apply more general and flexible imputation approaches.

Multiple imputation based on chained equations (MICE) has proven to be a useful imputation technique in different statistical applications (Clark & Altman, 2003; Ambler et al., 2007; van Buuren, 2007). This general approach is able to “rescue” other data that might otherwise be unusable, as a way to inform the imputation process in order to generate multiple imputed data sets upon which parameter estimation is then carried out. The final step of the analysis is to combine these imputed-based estimates and the respective standard errors in order to capture the underlying imputation uncertainty (Rubin, 1996). When first applied to genetic data, MICE produced unbiased estimates for genetic effects even when using imputation models not including auxiliary genetic information (Souverein et al., 2006). However, this first application was performed on European data that typically shows low genetic diversity together with long haplotypes and LD blocks (Conrad et al., 2006; Campbell & Tishkoff, 2008; Jakobsson et al., 2008). It is unclear whether MICE would have similar good performance in African data that often exhibits shorter LD patterns and haplotype blocks, due to high genetic diversity and/or population substructure.

This paper deals with genotype imputation in data from an association study (∼7000 individuals) of malaria parasite positivity in Tanzania (Drakeley et al., manuscript in preparation). We focus our study on the African α^3.7^-globin deletion, which occurs in the *HBA2* gene. The corresponding data set shows two unconventional characteristics. Firstly, the *HBA2* gene is not in strong LD with any other SNP in the data set. Thus, commonly used methods, such as BEAGLE, IMPUTE, or MACH, are not applicable to our data. Secondly, as a result of different sequencing efforts, there is an extremely high percentage of missing data on the α^3.7^-globin deletion (∼62%) in contrast to the low missing genotype percentage observed for the remaining SNPs in the data set (<10%). As a consequence, the subsequent statistical inferences will have different precision and power if only the complete case data are used. In this setting, MICE would appear to be the most promising tool to perform genotype imputation because one can inform the imputation process with genetic and nongenetic data. However, because of the high fraction of missing data on the α^3.7^-globin deletion, it is unclear whether this method could provide accurate results in terms of genetic association assessment, genetic effect estimation, and missing genotype prediction. The goal of this study was therefore to learn the limits of MICE in imputing missing genotypes in this nonstandard setting.

## Methods

### Study Population

The data set under analysis is part of a large cross-sectional study performed across 24 villages divided into six altitude transects (150–1800 m) in the Kilimanjaro and Tanga regions of Tanzania, as described elsewhere (Drakeley et al., 2005). The original sample size is about 7000 individuals with age between 6 months and 45 years old. Genotyping of the *HBA2* gene was only attempted in a subset of individuals living in 13 villages from four altitude-based transects (see Table 1). Considering individuals living in these 13 villages only, the sample size drops to 4414 individuals, for 36% of whom there is no information on the number of the α^3.7^-globin deletions.

**Table 1:** Background information of the 13 study sites where α^3.7^-globin genotyping was attempted

							No. of α^3.7^-globin deletions, n (%)					
			Major ethnic		Malaria parasite	Mild anemia						
Transect		Sample	group,	Females,	positivity,	prevalence,					Deletion	HWE
(region), village	Altitude, m	size, n	%	%	%	%	0	1	2	Missing	frequency	p-value
Kilimanjaro (Kilimanjaro)												
Mokala	1702	378	Wachaga (98.7)	61.9	4.5	14.8	154 (84.6)	27 (14.8)	1 (0.5)	196 (51.9)	0.080	0.387
Machame Aleni	1421	242	Wachaga (99.6)	54.5	1.7	9.0	168 (87.0)	25 (13.0)	0 (0.0)	49 (20.2)	0.065	0.188
Ikuini	1160	318	Wachaga (98.1)	60.3	10.7	19.9	157 (82.6)	32 (16.8)	1 (0.5)	128 (40.3)	0.089	0.235
Kileo	723	242	Wapare (84.3)	61.2	6.6	22.8	175 (75.1)	53 (22.7)	5 (2.1)	9 (3.7)	0.135	0.221
South Pare (Kilimanjaro)												
Bwambo	1598	375	Wapare (98.4)	57.3	3.2	20.5	178 (81.7)	37 (17.0)	3 (1.4)	157 (41.9)	0.099	0.504
Mpinji	1445	361	Wapare (95.0)	59.0	2.8	18.3	175 (75.1)	55 (23.6)	3 (1.3)	128 (35.5)	0.131	0.074
Goha	1162	389	Wapare (95.6)	60.3	10.9	20.1	172 (72.3)	62 (26.6)	4 (1.7)	151 (36.8)	0.147	0.047
Kadando	528	381	Wapare (70.5)	59.5	23.9	34.7	136 (57.9)	86 (36.6)	13 (5.5)	146 (38.3)	0.238	0.008
West Usambara (Tanga)												
Kwadoe	1523	404	Wasambaa (94.1)	61.6	7.7	33.4	166 (77.6)	46 (21.5)	2 (0.9)	190 (47.0)	0.117	0.106
Funta	1279	303	Wasambaa (97.0)	67.0	24.1	42.6	129 (61.1)	72 (34.1)	10 (4.7)	92 (30.4)	0.218	0.025
Tamota	1176	403	Wasambaa (93.5)	54.1	24.8	43.4	130 (58.6)	86 (38.7)	6 (2.7)	181 (44.9)	0.221	<0.001
Mgila	432	382	Wasambaa (67.7)	69.9	38.9	51.0	125 (54.8)	92 (40.4)	11 (4.8)	154 (40.3)	0.250	0.001
Tanga Coast (Tanga)												
Mgome	196	236	Other (86.3)	53.6	48.9	44.1	100 (43.7)	105 (45.9)	24 (10.5)	7 (3.0)	0.334	<0.001

### Phenotype and Genotype Data

Genotype, clinical and background data were generated as part of an ongoing larger project (http://www.malariagen.net; Drakeley et al., in preparation). In brief, genetic data refer to a total of 165 SNPs predominantly from malaria candidate genes (e.g., the sickle cell gene; *HbS*), of which 110 passed our stringent quality control step (minor allele frequency >5%, p-value for Hardy-Weinberg equilibrium in malaria-negative individuals >0.001, and missing data per SNP or individual >10%). A complete list of the SNPs can be found elsewhere for a similar study in Mali (Maiga et al., 2013).

We have also included data on the African α^3.7^-globin deletion that occurs in the *HBA2* gene located in the telomeric region of Chromosome 16 (16p13.3). Experimentally, the number of α^3.7^-globin deletions per individual genotype was determined by polymerase chain reaction assays as described elsewhere (Liu et al., 2000). Briefly, the PCR was carried out in a tetrad thermocycler (PTC-0240, The DNA engine Tetrad2® Thermal Cycler, Bio-Rad, Hercules, California, USA). DNA samples from individuals with known α^3.7^-globin deletion status (no deletion, one deletion, or two deletions) and samples with no DNA template as a negative control were included to each set of PCR reactions, as controls to assess the status of α^3.7^-globin deletion in the individuals. This was followed by gel electrophoresis in order to determine the number of α^3.7^-globin deletions (0, 1, or 2) defining the genotype of an individual.

For each individual in the study, we also have information on ethnicity, gender, age, hemoglobulin (Hb) levels, *Plasmodium falciparum* positivity (detected by microscopy inspection of blood smears on slides), and parasite density.

### Genetic Association Analysis

The purpose of the genetic association analysis is to determine whether the number of the α^3.7^-globin deletions affects *Plasmodium falciparum* positivity (hereafter referred to as malaria parasite positivity). We first performed an unadjusted association analysis between the number of α^3.7^-globin deletions and parasite positivity using the Pearson's χ^2^ test for two-way contingency tables. We then used logistic regression to test the genetic association, adjusting for putative socio-environmental confounders, such as “ethnicity” (Wapare, Wasambaa, Wachaga, and others), “age” (in years), “transect” (Kilimanjaro, South Pare, West Usambara, and Tanga coast) and “gender” (male/female). In this analysis, we compared two nested models, one including putative confounders only and another including both confounders and the genetic effects of the *HBA2* locus. In the latter model, we considered two independent genetic effects of the *HBA2* locus (λ_1_ and λ_2_ ), one associated with one deletion and another associated with two deletions. Model comparison was made by means of a likelihood ratio test, where a small p-value (e.g., <0.05) is indicative of a statistical association between the *HBA2* locus and malaria parasite positivity. Alternatively, one can use −log_10_(p-value) as a measure of association strength. Large values of this quantity suggest a strong association between the locus under analysis and the phenotype.

### Analysis of Missing Data

For each village where α^3.7^-globin genotyping was actually attempted, we tested whether missing genotypes were completely at random (MCAR) assuming a missing at random (MAR) mechanism as the saturated model for the data. This analysis was performed using the ACD package for the R software (Poleto et al., 2014).

We then used MICE to impute missing data under different scenarios and imputation models based on three-category logistic regression under a Multinomial sampling scheme (Souverein et al., 2006). In particular, we used the following logistic regression:

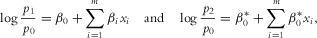
where *p_i_* is the probability of an individual having *i* α^3.7^-globin deletions, 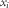 is the *i*th among *m* covariates and, 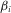 and 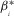 are the corresponding main effects. To construct the imputation model, we assessed the association between genetic and nongenetic variables and the number of α^3.7^-globin deletions, using the Pearson's χ^2^ test for two-way contingency tables or the likelihood ratio test for quantitative variables using polytomous logistic regression. MICE is based on the generation of *k* imputed data sets by the following algorithm: (1) generate random initial values for the missing genotypes, (2) estimate the above logistic regression model, (3) generate new random values for the original missing genotypes using the genotype probabilities predicted by the fitted model, (4) repeat steps 2 and 3 until convergence (of the chain), (5) estimate the corresponding genetic association model using the final iteration of the generated chain. To generate each imputed data set, we repeated the above algorithm with 25 or 100 iterations in order to have stable predictions for the missing data. For each (real or generated) data set, we generated 25 or 100 imputed data sets to capture the whole uncertainty underlying the imputation process.

After performing the association analysis in each imputed data set, one needs to combine the respective results into final estimates for the parameters of interest (Rubin, 1996). To this end, the genetic effects *λ_1_* and *λ_2_* were estimated by the mean of the respective postimputation estimates, that is,

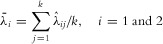
where 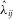 is the estimate of 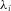 using the *j*-th imputed data set. The associated standard errors were given by

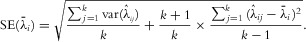
for a sufficiently large number of imputed data sets, the combined estimates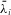 follow a Gaussian distribution approximately.

### Simulation Study

We carried out a simulation study in order to assess the performance of MICE on our data. We first adopted the standard approach of using the complete case data and treating a fraction of the existing genotypes as if they were missing, and attempted to impute them. We generated the missing genotypes independently of true α^3.7^-globin genotype (MCAR assumption). We generated 100 different random data sets with missing genotypes using three missing genotype proportions, 0.10, 0.25, and 0.50. Using the complete case data set again, we addressed a second situation where all genotypes were assumed missing from a given village.

To measure the performance of MICE using different imputation models, we compared different parametric inferences obtained from the complete case analysis (CCA) to those obtained after imputation. In particular, we calculated: (i) a pseudo estimation bias for the postimputation estimates of the average number of α^3.7^-globin deletions and the genetic effects for the phenotype-genotype association, (ii) a “pseudo coverage” of the postimputation 95% confidence intervals for the genetic effects, and (iii) the genotype error rate and r^2^ statistic to assess genotype imputation accuracy. We defined a “pseudo estimation bias” as the average difference between postimputation estimates and those obtained from CCA, while “pseudo coverage” was determined by the proportion of postimputation 95% confidence intervals that contained the genetic effects estimated from CCA. Genotype error rate was determined by the percentage of imputed genotypes in disagreement with the true ones. To estimate the average number of α^3.7^-globin deletions per individual, we used the following formula

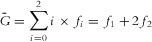
where *f_0_*, *f_1_*, and *f_2_* are the proportions of an individual having 0, 1, and 2 α^3.7^-globin deletions, respectively. In the case of the r^2^ statistic, we calculated the square of the correlation coefficient between the imputed and the true numbers of α^3.7^-globin deletions in the individuals with missing data, as done elsewhere (e.g., Li et al., 2011).

### Statistical Software

All statistical analyses (genetic association, imputation, and simulation) were carried out using the R software package (version 2.15). The corresponding scripts are available from the first author upon request.

## Results

### Tanzanian Villages Are Heterogeneous in Terms of Background, Clinical, and α^3.7^-Globin Deletion Data

Sample size, age, and gender are reasonably matched for the 13 villages where α^3.7^-globin genotyping was actually attempted (Table 1). Altitude varies from 1702 m in Mokala (Kilimanjaro) to 196 m in the coastal village of Mgome. In each transect, a specific ethnic group predominates: Wachaga in Kilimanjaro (with the exception of Kileo where Wapare is the main ethnic group), Wapare in South Pare, Wasambaa in West Usambara, and a mixed-ethnicity group in the Tanga coast. In the latter, the mixed-ethnicity group includes individuals of different ethnic groups but not the ones predominantly found in the other transects.

Villages are heterogeneous in terms of malaria parasite positivity and Hb levels. The overall malaria parasite positivity is 15.6%, ranging from 1.7% in the high-altitude village of Machame Aleni to 48.9% in the lowland of Mgome. The overall prevalence of mild anemia is 29.2% but varies considerably from village to village, where Machame Aleni and Mgila show the lowest and highest prevalence, 9.0% and 51.0%, respectively.

With respect to the number of α^3.7^-globin deletions per individual, the corresponding distribution differs among villages. The high-altitude villages of Mokala, Machame Aleni, Ikuini, and Bwambo show percentages of zero α^3.7^-globin deletions higher than 80%. On the other hand, 66% of the individuals from Mgome have at least one α^3.7^-globin deletion. The amount of missing data on α^3.7^-globin deletions varies with villages due to different genotyping efforts, ranging from 7 out of 236 individuals (3.0%) in Mgome to 196 out of 378 individuals (51.9%) in Mokala. The *HBA2* locus seems in Hardy-Weinberg equilibrium in most villages with the exception of Kadando, Tamota, Mgila, and Mgome where malaria tends to exert a higher selective pressure (Table 1).

### Association Analysis Using Complete Case Data

We started our association study by analyzing data only from individuals with no missing genotypes. The respective sample size decreased from 4414 to 2826 individuals. In this restricted data set, the average number of α^3.7^-globin deletions is around 0.337 (95% confidence interval: 0.317–0.357; Table 2). Unadjusted association analysis provided evidence for a strong effect of the α^3.7^-globin deletions on malaria parasite positivity (association signal: −log_10_(p-value) = 5.71). However, after adjusting for putative confounders, the association signal dropped to 0.80 (Table 2). This analysis suggests that the number of α^3.7^-globin deletions is not associated with malaria parasite positivity, a result in opposition to the strong association signals for severe malarial anemia in Tanzanian children (Manjurano et al., 2012) and clinical phenotypes in Kenya (Williams, Wambua et al., 2005).

**Table 2:** Association analysis between the number of α^3.7^-globin deletions and malaria parasite positivity using complete case data. “Log-likelihood” refers to the maximum value of the log-likelihood function after maximum likelihood parameter estimation. Association analysis was performed adjusting or not for putative confounders (age, gender, ethnicity, altitude, and transect). Association signals refer to −log_10_(p-value), where p-value is from Pearson's χ^2^ test for two-way contingency tables in the unadjusted analysis, and from the likelihood ratio test for lack of genetic association in the adjusted analysis

	Unadjusted analysis		Adjusted analysis	
Parameter	Estimate	SE	Estimate	SE
Average no. of deletions	0.337	0.010	−	−
λ_1_	0.408	0.121	−0.225	0.138
λ_2_	1.165	0.250	0.179	0.283
Log-likelihood	−1069.16	−	−876.99	−
Association signal	5.71	−	0.80	

### Missing Genotypes Are Generated by Different Random Mechanisms across Villages

**Figure 1:**
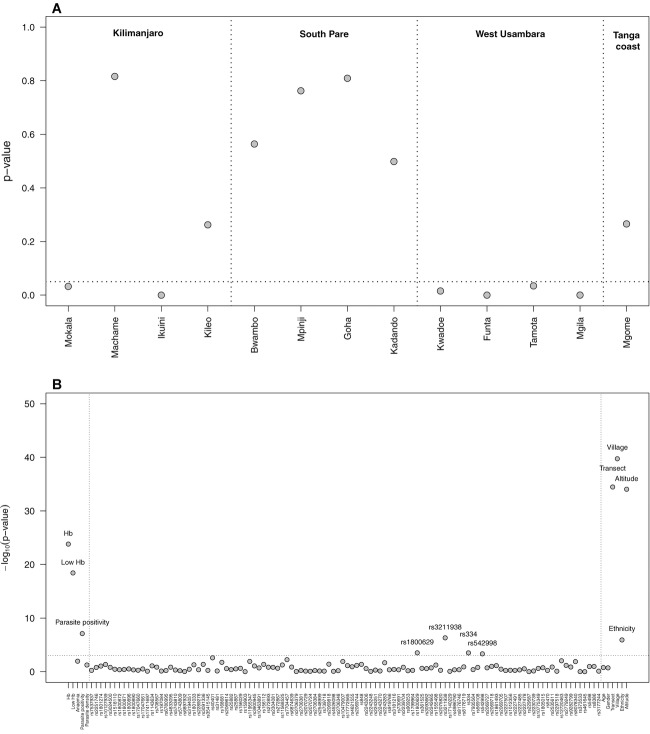
(A) Testing a missing completely at random (MCAR) hypothesis under the basic assumption of the missing at random (MAR) model for the data resulting from the cross-tabulation of the number of α^3.7^-globin deletions with malaria parasite positivity. Each dot represents the p-value for the corresponding likelihood ratio test. Horizontal pointed line refers to the 5% significance level. In this analysis, we accepted the MCAR hypothesis on data from villages where p-value >0.05. The rejection of MCAR led to the acceptance of an MAR mechanism. (**B)** Association analysis between α^3.7^-globin deletions and different variables (phenotypes – at the left, SNPs – at the centre, and socioenvironmental factors – at the right) using complete data. Association signal is expressed in terms of −log_10_(p-value) for the corresponding association test: χ^2^ test for categorical explanatory variables (SNPs, low Hb, anemia, parasite positivity, gender, transect, village, and ethnicity) and score tests for quantitative explanatory variables (Hb levels, parasite density, age, and altitude) using a three-category logistic regression framework. Horizontal dashed line refers to −log_10_(0.001) corresponding to a 0.1% significance level.

Before performing data imputation per se, we first tested whether missing data from each village would follow either an MCAR or an MAR mechanism (Fig. 1A). Overall, there are 7 of 13 villages where an MCAR mechanism holds at the 5% significance level. Missing data from the remaining villages are instead compatible with an MAR mechanism. Curiously, an MAR mechanism holds on data from the four villages in the West Usambara transect, whereas an MCAR is a reasonable assumption for data from villages in the South Pare transect. A mixture of MCAR and MAR mechanisms is found for data of villages from the Kilimanjaro transect. Therefore, different efforts in genotyping the *HBA2* gene across study sites resulted in distinct missing data mechanisms for the data.

### Building the Imputation Models for MICE

Motivated by the high frequency of missing data for the α^3.7^-globin deletions (1588 out of 4414), we studied the performance of MICE in estimating different parameters of interest. Using the subset of 2826 individuals with complete information on α^3.7^-globin deletion status, we conducted a preliminary association analysis between that variable and the remaining data in order to identify the most informative covariates for the imputation process. In this analysis, we had a total of 110 SNPs that passed our stringent quality control step, five phenotypes measured in the individuals, and five socioenvironmental factors (Fig. 1B). There are only four SNPs strongly associated with the number of α^3.7^-globin deletions: rs1800629 (*TNFα*, chr. 6), rs3211938 (*CD36*, chr. 7), rs334 (*HbS*, chr. 11), and rs542998 (*RTN3*, chr. 11). The association between HbS and α^3.7^-globin deletions was previously detected in the same data set by observing a higher frequency of heterozygous individuals in both genes than that expected under independent segregation (Enevold et al., 2007). In contrast, a weak LD between these two loci was previously detected in the neighboring country Kenya (Williams, Mwangi et al., 2005). In addition to these four SNPs, missing data may also be informed from the following covariates: Hb levels, mild anemia and malaria parasite positivity, and transect. These eight data variables were then used to construct three imputation models with the following covariates: (i) rs1800629, rs3211938, rs334, and rs542998 (model IM_1_); (ii) Hb levels, mild anemia positivity, malaria parasite positivity, and transect (model IM_2_), and (iii) all covariates included in previous models (model IM_3_). We studied the performance of each model and compared them to the simple multiple imputation procedure based on the observed frequency of α^3.7^-globin deletions (model IM_0_).

### Imputation Models IM_2_ and IM_3_ Led to the Smallest Decrease in the Association Signals When Missing Data Are Completely at Random

We first studied the performance of MICE simulating missing data under an MCAR mechanism. The estimated genotype error rates are ∼44% irrespective of the imputation model used, including IM_0_, and the amount of missing genotypes in the data set (Table 3). In line with this result, we also found a low value of the r^2^ statistic on average (r^2^ < 0.10; Table S1). Therefore, in the absence of SNPs in strong LD with the *HBA2* gene, MICE has limited power in predicting the missing number of α^3.7^-globin deletions with great precision. Notwithstanding this limitation, all imputation models could provide unbiased estimates for the average number of α^3.7^-globin deletions. The range of these estimates increased with the missing data proportion.

**Table 3:** Genotype-based performance of different imputation models: IM_0_ refers to imputation carried out using the observed frequencies of α^3.7^-globin deletions, IM_1_ includes four SNPs as imputation covariates (rs1800629, rs3211938, rs334, and rs542998), IM_2_ includes eight phenotypes and socio-environmental factors (Hb, mild anemia, malaria parasite positivity, transect, altitude, and ethnicity), and IM includes all variables in IM and IM

	IM_0_		IM_1_		IM_2_		IM_3_	
Missing completely	Genotype error	Average no. of	Genotype error	Average no. of	Genotype error	Average no. of	Genotype error	Average no. of
at random^a^	rate (range), %	deletions (range)	rate (range), %	deletions (range)	rate (range), %	deletions (range)	rate (range), %	deletions (range)
P_miss_ = 10%	44.1 (41.1–47.0)	0.34 (0.33–0.35)	44.1 (41.1–47.3)	0.337 (0.33–0.35)	44.3 (40.8–47.4)	0.34 (0.33–0.35)	44.1 (41.0–47.5)	0.34 (0.33–0.35)
P_miss_ = 25%	44.3 (41.9–46.1)	0.34 (0.33–0.35)	44.2 (41.7–46.3)	0.338 (0.33–0.35)	44.3 (41.9–46.3)	0.34 (0.32–0.35)	44.4 (41.8–46.9)	0.34 (0.32–0.35)
P_miss_ = 50%	44.2 (42.8–45.8)	0.34 (0.31–0.37)	44.1 (42.5–45.9)	0.337 (0.31–0.37)	44.2 (42.5–45.9)	0.34 (0.31–0.37)	44.3 (42.6–45.9)	0.34 (0.31–0.37)
Missing data from one village^b^								
Kilimanjaro								
Mokala	37.7 (28.9–47.4)	0.35 (0.34–0.36)	36.4 (27.7–47.4)	0.38 (0.33–0.36)	26.8 (20.8–32.9)	0.35 (0.34–0.35)	26.5 (19.1–35.8)	0.32 (0.32–0.33)
Machame	37.1 (28.6–46.9)	0.35 (0.35,0.36)	37.1 (28.6–46.3)	0.36 (0.36–0.38)	27.6 (21.1–34.9)	0.38 (0.37–0.38)	26.8 (18.3–34.3)	0.36 (0.36–0.37)
Ikuini	38.6 (27.7–45.1)	0.35 (0.34,0.36)	36.9 (27.7–48.9)	0.35 (0.34–0.36)	30.5 (30.5–22.8)	0.32 (0.32–0.33)	29.8 (22.8–38.6)	0.33 (0.33–0.34)
Kileo	41.8 (34.6–49.6)	0.34 (0.34,0.35)	40.9 (32.0–47.4)	0.34 (0.33–0.36)	38.7 (28.1–53.5)	0.34 (0.32–0.38)	36.1 (28.1–49.6)	0.35 (0.34–0.38)
South Pare								
Bwambo	39.1 (32.2–45.8)	0.35 (0.34,0.36)	37.3 (29.9–44.4)	0.35 (0.34–0.36)	32.2 (25.7–40.7)	0.34 (0.33–0.35)	31.8 (24.3–39.7)	0.34 (0.33–0.35)
Mpinji	41.5 (32.5–49.6)	0.34 (0.34,0.36)	41.0 (31.6–47.8)	0.35 (0.34–0.37)	34.6 (26.8–40.8)	0.34 (0.34–0.35)	37.4 (26.3–45.6)	0.33 (0.33–0.33)
Goha	43.0 (35.7–48.7)	0.34 (0.33, 0.35)	42.8 (36.6–49.1)	0.34 (0.33–0.35)	39.4 (31.7–46.0)	0.32 (0.32–0.33)	40.5 (32.1–46.4)	0.33 (0.33–0.34)
Kadando	49.6 (41.9–59.0)	0.33 (0.32, 0.33)	49.2 (40.1–55.8)	0.34 (0.33–0.35)	52.0 (45.2–60.4)	0.33 (0.32–0.34)	51.6 (45.2–59.4)	0.34 (0.33–0.35)
West Usambara								
Kwadoe	40.1 (31.5–46.9)	0.35 (0.34, 0.35)	40.2 (33.8–48.4)	0.35 (0.34–0.37)	39.0 (31.9–46.5)	0.37 (0.36–0.38)	38.9 (30.5–45.1)	0.36 (0.36–0.37)
Funta	47.6 (41.3–56.8)	0.33 (0.32,0.34)	48.6 (41.3–56.8)	0.34 (0.33–0.35)	47.3 (38.8–52.9)	0.33 (0.32–0.33)	48.3 (39.8–56.3)	0.33 (0.33–0.34)
Tamota	48.8 (41.4–55.8)	0.33 (0.32,0.34)	48.1 (41.4–55.3)	0.34 (0.34–0.36)	48.4 (43.3–56.7)	0.34 (0.33–0.34)	48.7 (40.9–58.1)	0.36 (0.36–0.37)
Mgila	51.3 (43.8–58.1)	0.32 (0.32,0.33)	50.7 (43.8–56.7)	0.35 (0.35–0.37)	53.3 (43.3–60.6)	0.39 (0.38–0.40)	55.0 (46.8–63.1)	0.37 (0.36–0.39)
Tanga coast								
Mgome	57.3 (50.2–66.7)	0.31 (0.30,0.32)	55.9 (48.9–64.4)	0.33 (0.32–0.35)	56.6 (47.0–67.1)	0.35 (0.32–0.40)	55.2 (46.6–65.8)	0.35 (0.32–0.42)

a Results based on 100 MCAR data sets in which each data set was analyzed by MICE using 25 imputed data sets generated from chains of 25 iterations and random initial conditions.

b Results based on 100 imputed data sets generated by MICE using chains of 25 iterations and random initial conditions.

With respect to the association signals, the different imputation models led to an overall decrease in the maximum value of the log-likelihood, irrespective of the missing data proportion (Table 4). This decrease might be explained by the absence of additional covariates from the imputation models. A direct consequence of this result is a weakening of the association signal in relation to that of the CCA. IM_2_ and IM_3_ led to little estimation bias (0.11%–0.13%) of the maximum of the CCA log-likelihood function. Although producing genetic effect estimates with least bias, IM_0_ and IM_1_ led to the largest estimation bias (∼1%) of the maximum of the CCA log-likelihood function. Finally, every imputation model could generate confidence intervals that all included the genetic effect estimates obtained from CCA. This result indicates that there is a high degree of uncertainty underlying the genetic effect estimation.

**Table 4:** Performance of different imputation models in terms of genetic effect estimation: IM_0_ refers to imputation carried out using the observed frequencies of the α^3.7^-globin deletions, IM_1_ includes four SNPs as imputation covariates (rs1800629, rs3211938, rs334, and rs542998), IM_2_ includes eight phenotypes and socio-environmental factors (Hb, mild anemia, malaria parasite positivity, transect, altitude, and ethnicity), and IM_3_ includes all variables in IM_1_ and IM_2_

	IM_0_			IM_1_			IM_2_			IM_3_		
		Estimation bias			Estimation bias			Estimation bias			Estimation bias	
	Log-	(CI coverage, %)		Log-	(CI coverage, %)		Log-	(CI coverage, %)		Log-	(CI coverage, %)	
	likelihood			likelihood			likelihood			likelihood		
Missing completely at random^a^	bias (%)	λ_1_	λ_2_	bias (%)	λ_1_	λ_2_	bias (%)	λ_1_	λ_2_	bias (%)	λ_1_	λ_2_
P_miss_ = 10%	−8.80 (1.00)	0.04 (100)	−0.06 (100)	−8.80 (1.00)	0.03 (100)	−0.06 (100)	−1.16 (0.13)	0.10 (100)	−0.07 (100)	−1.14 (0.13)	0.10 (100)	−0.08 (100)
P_miss_ = 25%	−8.90 (1.02)	0.07 (100)	−0.04 (100)	−8.95 (1.02)	0.07 (100)	−0.06 (100)	−1.02 (0.12)	0.11 (100)	−0.09 (100)	−1.00 (0.11)	0.10 (100)	−0.10 (100)
P_miss_ = 50%	−9.12 (1.04)	0.12 (100)	−0.09 (100)	−9.16 (1.05)	0.12 (100)	0.13 (100)	−0.93 (0.11)	0.13 (100)	−0.15 (100)	−0.93 (0.11)	0.13 (100)	−0.16 (100)
Missing data from one village^b^												
Kilimanjaro												
Mokala	−0.19 (0.02)	0.02 (100)	0.01 (100)	−1.47 (0.17)	0.24 (80)	0.13 (100)	7.83 (0.89)	−0.31 (8)	0.41 (100)	2.63 (0.30)	0.19 (100)	0.64 (0)
Machame	−0.06 (0.01)	0.01 (100)	<−0.01 (100)	−0.90 (0.11)	0.06 (100)	−0.08 (100)	4.39 (0.50)	−0.16 (100)	0.25 (100)	1.62 (0.19)	−0.02 (100)	0.20 (100)
Ikuini	0.21 (0.02)	−0.02 (100)	−0.01 (100)	−0.80 (0.09)	0.07 (100)	−0.01 (100)	1.20 (0.13)	−0.09 (100)	−0.05 (100)	0.51 (0.06)	−0.02 (100)	0.07 (100)
Kileo	0.46 (−0.05)	−0.05 (100)	−0.07 (100)	−0.19 (0.02)	0.00 (100)	−0.05 (100)	2.71 (0.31)	−0.15 (100)	0.05 (100)	1.98 (0.23)	−0.07 (100)	0.14 (100)
South Pare												
Bwambo	−0.30 (0.03)	0.01 (100)	−0.07 (100)	−0.30 (0.03)	0.01 (100)	−0.07 (100)	−0.26 (0.03)	0.01 (100)	−0.06 (100)	−0.25 (0.02)	0.01 (100)	−0.06 (100)
Mpinji	0.21 (−0.02)	−0.02 (100)	−0.01 (100)	−1.50 (0.17)	0.22 (90)	0.10 (100)	8.66 (0.99)	−0.34 (0)	0.42 (100)	2.80 (0.32)	0.16 (100)	0.64 (0)
Goha	−0.15 (0.03)	0.03 (100)	0.04 (100)	−0.46 (0.05)	0.03 (100)	−0.05 (100)	1.07 (0.12)	−0.08 (100)	−0.03 (100)	0.26 (0.26)	0.01 (100)	0.10 (100)
Kadando	−0.33 (0.04)	0.06 (100)	0.07 (100)	−0.94 (0.11)	0.11 (100)	0.03 (100)	1.27 (0.15)	−0.06 (100)	0.06 (100)	1.14 (0.13)	0.01 (100)	0.22 (100)
West Usambara												
Kwadoe	0.02 (<0.01)	<−0.01 (100)	−0.02 (100)	−0.20 (0.02)	−0.00 (100)	−0.08 (100)	4.05 (0.46)	−0.10 (100)	0.35 (99)	2.73 (0.31)	−0.08 (100)	0.20 (100)
Funta	0.04 (<0.01)	0.02 (100)	0.05 (100)	−0.30 (0.03)	0.02 (100)	−0.04 (100)	0.00 (0.00)	0.00 (100)	−0.02 (100)	0.12 (0.01)	−0.01 (100)	−0.03 (100)
Tamota	−0.66 (0.07)	0.05 (100)	−0.05 (100)	−1.60 (0.18)	0.25 (62)	−0.02 (100)	9.63 (1.10)	−0.38 (4)	0.38 (96)	2.74 (0.31)	0.23 (83)	0.65 (13)
Mgila	0.24 (0.03)	0.07 (100)	0.21 (100)	−0.65 (0.07)	0.13 (100)	0.16 (100)	8.56 (0.98)	−0.21 (87)	0.47 (58)	4.35 (0.50)	−0.02 (100)	0.43 (70)
Tanga coast												
Mgome	−0.75 (0.09)	0.04 (100)	−0.32 (100)	−1.32 (0.15)	0.18 (88)	0.05 (100)	8.15 (0.93)	−0.33 (23)	0.41 (94)	3.38 (0.39)	0.10 (100)	0.59 (8)

a Results based on 100 MCAR data sets in which each data set was analyzed by MICE using 25 imputed data sets generated from chains of 25 iterations and random initial conditions.

b Results based on 100 imputed data sets generated by MICE using chains of 25 iterations and random initial conditions.

### MICE May Show Estimation Bias If All Missing Genotypes Were Assumed to Come from the Same Study Site

We then studied the performance of MICE in a scenario where the genotyping of the *HBA2* gene was not attempted in individuals from a given village. The genotype error rate varies with imputation models and villages with missing genotypes (Table 3). The lowest genotype error rate was obtained from both IM_2_ and IM_3_ (∼40%; 44.3% – IM_0_, 43.7% – IM_1_). Similar qualitative conclusion can be taken from the r^2^ statistic (Table S1). In these two models, the lowest and highest genotype errors were obtained from data of Mokala in the Kilimanjaro transect (genotype error rates: 26.8% – IM_2_, 26.5% – IM_3_) and Mgome in the Tanga coast (56.6% – IM_2_, 55.2% – IM_3_), respectively. Conversely, the highest value for the r^2^ statistic was found for Kileo data (0.015 – IM_2_ and 0.017 – IM_3_) with the exception of the IM_2_ for the Mokala data (r^2^ = 0.20). Curiously, each imputation model could perform better in missing data from villages in the Kilimanjaro transect than in the others. The reason for this is unclear but it might be related to a stronger association of the α^3.7^-globin deletions with the phenotypes and/or socio-environmental factors in this specific transect. On the other hand, all imputation models produced a very high genotype error rate for the missing data from Mgome (>55%). This result can be explained by a different selective pressure in this village due to a higher malaria exposure (Table 1).

Using IM_1_, IM_2_, and IM_3_, there is an inverse correlation between altitude and genotype error rates. Missing genotypes tend to be better predicted in high-altitude villages than in those at the lowlands of the respective transects. An example of this is the West Usambara transect where the genotype error rate varies from 31.8% in Bwambo, a high-altitude village, to 51.6% in Kadando in the lowlands of that transect. This result is in line with an increasing number of zero α^3.7^-globin deletions from the lowlands to high-altitude sites where the malaria positivity rates are much lower (Table 1).

With respect to the average number of α^3.7^-globin deletions, it is difficult to ascertain the imputation model with the best performance (Table 3). The corresponding estimates can be biased or unbiased, depending on the source of the missing data. On the one hand, one can obtain biased estimates when missing data come from Machame Aleni, even if the underlying genotype error rate is reasonably low (26.8%, IM_3_). On the other hand, irrespective of the imputation model used, little bias was obtained when missing data were from villages in the West Usambara transect. Imputing genotypes of Mgome, although leading to a high genotype error rate, did not lead to an extremely high estimation bias.

The simple imputation procedure based on genotype proportions (IM_0_) led to approximately unbiased association signals and genetic effect estimates, irrespective of the missing data under analysis (Table 4). In contrast, IM_1_ implied an overall weakening of the association signals by decreasing the maximum of the CCA log-likelihood function by 0.2%. The corresponding genetic effect estimates are approximately unbiased with the exception of *λ_1_* when missing data came from Tamota. Both IM_2_ and IM_3_ tend to strengthen the association signals by increasing the maximum of the CCA log-likelihood function. The highest increase in the association signal is observed for IM_2_ when missing data came from Tamota (∼1.1% inflation of the maximum of the CCA log-likelihood function). The corresponding genetic effect estimates are in most cases unbiased with a few exceptions. IM_2_ seems to lead to biased estimates for λ_1_ when missing data came from Mokala (Kilimanjaro), Mpinji (South Pare), Tamota (West Usambara), or Mgome (Tanga coast). Conversely, IM_3_ tends to produce biased estimates of λ_2_.

### Imputation of Truly Missing Data from Lowland Villages Significantly Increased the Association Signals

We finally performed genotype imputation on truly missing data and the corresponding association analysis. Table S2 shows the background information for the 11 villages where the genotyping of the *HBA2* locus was not actually attempted. In brief, these data seem to be fairly matched for key socio-environmental factors and, more importantly, we can still find an inverse correlation between malaria parasite positivity and altitude.

We performed three different association analysis: (i) imputation of missing data from the 13 villages studied thus far (*scenario* 1), (ii) imputation of missing data from the same 13 villages and an additional village where genotyping of the *HBA2* gene was not attempted (*scenario* 2), and (iii) imputation of missing data from the 24 villages included in the original study design (*scenario* 3). According to our simulation study, we considered IM_3_ as the best imputation model for the data because it appears to produce a good compromise between estimation and association bias. The remaining results for IM_0_, IM_1_, and IM_2_ can be found in Table S3.

In the case of scenario 1, the average number of α^3.7^-globin deletions decreased from 0.337 (Table 2; CCA) to 0.326 (Table 5; after imputation). In turn, the association signal increased from 0.80 (Table 2) to 1.47 (Table 5). The estimates of the underlying genetic effects are similar but the respective standard errors are now smaller (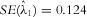 and 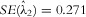 after imputation vs. 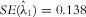 and 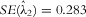 in CCA). Therefore, a stronger association signal seems a direct effect of increasing the sample size.

**Table 5:** Genetic association analysis using IM_3_ (100 imputed data sets) under different data settings: (i) data of 13 villages where genotyping of the *HBA2* gene was attempted in the majority of the individuals, (ii) data of the same 13 villages and an additional village where geno-typing was not attempted, and (iii) all data from the 24 villages

					Estimates (SE)	
	Total sample	Missing	Mean association	Average no.		
Analysis	size, n	genotypes, %	signal (range)^a^	of deletions	λ_1_	λ_2_
13 villages^b^	4143	34.9	1.47 (0.42–3.22)	0.326 (0.01)	−0.226 (0.12)	0.181 (0.27)
13 villages and an additional village^b^						
North Pare						
Kilomeni	4433	39.1	1.47 (0.19–3.68)	0.32 (0.01)	−0.22 (0.13)	0.19 (0.270)
Lambo	4405	38.7	1.59 (0.18–4.28)	0.32 (0.01)	−0.24 (0.13)	0.18 (0.27)
Ngulu	4499	40.0	1.62 (0.29–4.75)	0.33 (0.01)	−0.23 (0.13)	0.17 (0.28)
Kambi ya Simba	4356	38.0	1.65 (0.13–4.42)	0.33 (0.01)	−0.23 (0.13)	0.20 (0.26)
West Usambara 1						
Emmao	4321	37.5	1.51 (0.20–3.98)	0.33 (0.01)	−0.23 (0.13)	0.17 (0.27)
Handei	4489	39.9	1.67 (0.38–4.87)	0.33 (0.01)	−0.23 (0.12)	0.21 (0.27)
Tewe	4463	39.5	1.88 (0.21–5.21)	0.33 (0.01)	−0.23 (0.13)	0.20 (0.27)
Mn'galo	4490	39.9	1.75 (0.27–4.56)	0.33 (0.01)	−0.22 (0.13)	0.21 (0.27)
West Usambara 2						
Magamba	4351	38.0	1.56 (0.59–3.51)	0.32 (0.01)	−0.23 (0.12)	0.20 (0.27)
Ubiri	4297	37.2	1.77 (0.07–5.08)	0.33 (0.01)	−0.25 (0.13)	0.17 (0.27)
Kwemasimba	4374	38.3	1.83 (0.21–4.63)	0.34 (0.01)	−0.24 (0.13)	0.18 (0.27)
24 villages^c^	7048	61.7	2.70 (0.10–6.75)	0.34 (0.03)	−0.23 (0.12)	0.18 (0.25)

a Association signal is calculated by −log_10_(p-value) using either the mean or the median of log-likelihood ratio statistic across all imputed data sets.

b Results of model IM_3_ were obtained from imputed data using chains of 25 iterations and random initial conditions.

c Results of model IM_3_ were obtained from imputed data using chains of 100 iterations and random initial conditions.

With respect to scenario 2, the overall missing genotype percentage varies from 37.2% (Ubiri) to 40.0% (Ngulu). The estimates of the average number of α^3.7^-globin deletions are similar to the ones from previous imputation scenario, irrespective of the village added into the analysis with the exception of Kwemasimba (average number of α^3.7^-globin deletions = 0.337). The mean association signals, ranging from 1.48 (Kilomeni) to 1.88 (Tewe), are inversely correlated with altitude across villages of the same transect. Genetic effect estimates range from −0.246 (Ubiri) to −0.216 (Handei) for *λ_1_* and from 0.165 (Ubiri) to 0.208 (Handei) for *λ_2_*, respectively.

When we analysed the whole data set of the 24 villages (n = 7048) where missing genotypes is close to 62%, the average number of α^3.7^-globin deletions was estimated to be 0.336 after imputation (Table 5), a value in line with that from CCA but associated with a higher standard error (*SE* = 0.010 and 0.034 before and after imputation, respectively). The post imputation association signals span more than six orders of magnitude (from 0.10 to 6.75) with an average of 2.70. The genetic effect estimates for *λ_1_* and *λ_2_* are −0.232 (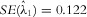) and 0.183 (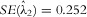), respectively. These estimates, though associated with smaller standard errors, do not differ substantially from those obtained from CCA. This result suggests that the imputation is capturing the same genetic effects produced by CCA but increasing estimation precision due to a larger number of individuals included in the analysis.

## Discussion

This study concerns MICE and its utility in imputing missing data from Tanzania on the α^3.7^-globin deletion. We showed that, when missing genotypes were simulated under an MCAR assumption, MICE using model IM_3_ led to genotypic error rates of ∼44% and low r^2^ statistics but unbiased estimates for the different genetic parameters and association signals. We then conclude that MICE is useless in predicting the true status of the *HBA2* gene of a particular individual but is rather useful in providing reliable genetic effect estimates and association signals. In theory, we expect that MICE would show better genotype prediction accuracy if we had in our data set any genetic marker in strong LD with the *HBA2* gene. In line with this expectation is the study of Souverein et al. (2006) where MICE, when applied to linked genetic marker data, led to genotype error rates <20%. However, it is still unclear whether MICE using an appropriate imputation model is capable of decreasing the genotype error rates to figures close to those observed in GWAS (∼5%–6% on average; Marchini & Howie, 2010), thus allowing the performance of accurate haplotype analysis. Previous studies have shown that the genotype prediction accuracy is dependent on the number of strongly linked SNPs to the one with missing information, the amount of missing data, the population under analysis, and/or reference panel, if any is used to inform the imputation process (Pei et al., 2008; Huang et al., 2009; Nothnagel et al., 2009; Chanda et al., 2012; Hancock et al., 2012). Additionally, genotype error rates in MICE are dependent on the imputation model used for the analysis, as demonstrated in our simulation study. Since MICE has the possibility of including genetic and nongenetic information in the imputation process, we do not expect any theoretical impediment of this framework in predicting missing genotypes more accurately.

We also assessed the performance of MICE assuming that the genotyping of the *HBA2* gene was not attempted in individuals from a given village. Since different combinations of socio-environmental factors characterize the villages in this study, the resulting missing data might be dependent on specific characteristics of the individuals of these villages, thus leading to a putative missing not at random (MNAR) mechanism. In this case, MICE using model IM_3_ can lead to biased or unbiased genetic effect estimates and association signals, depending on the villages where missing data comes from. The source of this bias is not totally clear but it may reflect the “strength” of an MNAR mechanism for the data. In fact, our results showed little bias while analyzing missing data from villages in intermediate altitudes because imputation can borrow information from other villages characterized by similar genetic and socio-environmental factors. Conversely, there is a significant bias when missing data comes from the coastal village of Mgome, which exhibits unique characteristics among all the 13 villages used in our simulation study. Thus, MICE under a putative MNAR mechanism should be used with caution, as also alerted by Souverein et al. (2006).

The possibility of producing biased estimates when missing data comes from a specific village poses the question about the reliability and robustness of the results for the whole data set where there is no α^3.7^-globin data for entire transects (North Pare and West Usambara 1 and 2). In this case, it is expected that missing genotypes are the result of a mixture between MCAR, MAR, and MNAR mechanisms. MAR or MCAR might arise from data of villages where genotyping of the *HBA2* gene was actually attempted, while MNAR might occur in data from villages where genotyping of that gene was not attempted. In this overall analysis, the association signals of the α^3.7^-globin deletions became statistically significant, most likely due to an increased number of individuals under the analysis. If true, the use of MICE, besides increasing estimation precision, seems invaluable in detecting additional association signals from known and unknown genes with smaller effect that otherwise would remain undetected. This is particularly relevant in association studies of nonclinical malaria phenotypes where different genes with only moderate effects could be detected, as demonstrated by recent data from Mali (Maiga et al., 2013).

We believe that, under the realistic assumption of similar genetic pressure across transects, our results for the whole data set show no strong bias as a result of our well-balanced study design. The use of transects and their relationship with the high prevalence of specific ethnic groups was invaluable in controlling putative genetic substructures in the study area. A design using altitude-based transects has ensured the comparability of the villages in terms of malaria parasite exposure. The additional matching for gender and age has minimized further selective bias. The key to minimal estimation bias after data imputation therefore relies on having an adequate study design.

In conclusion, MICE using an appropriate imputation model shows limitations in predicting the missing data of the α^3.7^-globin deletions of a given individual, but seems to lead to the correct genetic effect estimates and association signals as long as the data are well-matched for key socio-environmental factors.

## Supporting Information

**Table S1** Mean genotype accuracy (range in brackets) using the squared correlation between the imputed and true number of α^3.7^-globin deletions, where IM_0_ refers to imputation carried out using the observed frequencies of α^3.7^-globin deletions, IM_1_ includes four SNPs as imputation covariates (rs1800629, rs3211938, rs334, and rs542998), IM_2_ includes eight phenotypes and socioenvironmental factors (Hb, mild anemia, malaria parasite positivity, transect, altitude, and ethnicity), and M_3_ includes all variables in M_1_ and M_2_. **Table S2** Background information of the 11 villages where α^3.7^-globin genotyping was not attempted. **Table S3** Association analysis after carrying out genotype imputation by means of IM_0_ and IM_1_ using data from: (i) the 13 villages where α^3.7^-globin genotyping was attempted in the majority of the individuals, (ii) the same 13 villages and an additional village where α^3.7^-globin genotyping was not attempted, and (iii) all the 24 villages.
