# Supplementary material for: On the Performance of Multiple Imputation Based on Chained Equations in Tackling Missing Data of the African α3.7-Globin Deletion in a Malaria Association Study
Source: Ann Hum Genet. 2014 Jun 18;78(4):277–89. doi: 10.1111/ahg.12065 (PMC4140543; doi:10.1111/ahg.12065)
Supplement: Supplementary file 1 — Table S1 Mean genotype accuracy (range in brackets) using the squared correlation between the imputed and true number of α3.7-globin deletions, where IM0 refers to imputation carried out using the observed frequencies of α3.7-globin deletions, IM1 includes four SNPs as imputation covariates (rs1800629, rs3211938, rs334, and rs542998), IM2 includes eight phenotypes and socioenvironmental factors (Hb, mild anemia, malaria parasite positivity, transect, altitude, and ethnicity), and M3 includes all variables in M1 and M2. Table S2 Background information of the 11 villages where α3.7-globin genotyping was not attempted. Table S3 Association analysis after carrying out genotype imputation by means of IM0 and IM1 using data from: (i) the 13 villages where α3.7-globin genotyping was attempted in the majority of the individuals, (ii) the same 13 villages and an additional village where α3.7-globin genotyping was not attempted, and (iii) all the 24 villages. [file ahg0078-0277-sd1.docx]

**Supplementary Table 1**

Mean genotype accuracy (range in brackets) using the squared correlation between the imputed and true number of α^3.7^-globin deletions, where IM_0_ refers to imputation carried out using the observed frequencies of α^3.7^-globin deletions, IM_1_ includes 4 SNPs as imputation covariates (rs1800629, rs3211938, rs334, and rs542998), IM_2_ includes 8 phenotypes and socio-environmental factors (Hb, mild anaemia, malaria parasite positivity, transect, altitude, and ethnicity), and M_3_ includes all variables in M_1_ and M_2_.

| Simulation scenario | | | IM_0_ | IM_1_ | IM_2_ | IM_3_ |
| --- | --- | --- | --- | --- | --- | --- |
| Missing completely at random^a^ | | |  |  |  |  |
|  | P_miss_=10% | | 0.004 (0.001-0.009) | 0.005 (0.002-0.009) | 0.014 (0.006-0.029) | 0.016 (0.004-0.027) |
|  | P_miss_=25% | | 0.001 (0.001-0.003) | 0.002 (0.001-0.004) | 0.012 (0.006-0.021) | 0.014 (0.008-0.023) |
|  | P_miss_=50% | | 0.001 (0.000-0.001) | 0.001 (0.001-0.002) | 0.011 (0.008-0.015) | 0.013 (0.009-0.017) |
| Missing data from one village^b^ | | |  |  |  |  |
|  | Kilimanjaro | |  |  |  |  |
|  |  | Mokala | 0.005 (0-0.056) | 0.005 (0-0.025) | 0.020 (0-0.108) | 0.010 (0-0.107) |
|  |  | Machame | 0.005 (0-0.030) | 0.006 (0-0.037) | 0.005 (0-0.038) | 0.006 (0-0.037) |
|  |  | Ikuini | 0.005 (0-0.046) | 0.006 (0-0.042) | 0.006 (0-0.040) | 0.008 (0-0.064) |
|  |  | Kileo | 0.004 (0-0.025) | 0.006 (0-0.046) | 0.015 (0-0.047) | 0.017 (0-0.082) |
|  | South Pare | |  |  |  |  |
|  |  | Bwambo | 0.004 (0-0.040) | 0.005 (0-0.036) | 0.004 (0-0.033) | 0.008 (0-0.069) |
|  |  | Mpinji | 0.004 (0-0.036) | 0.006 (0-0.051) | 0.005 (0-0.037) | 0.006 (0-0.097) |
|  |  | Goha | 0.005 (0-0.026) | 0.005 (0-0.032) | 0.004 (0-0.031) | 0.005 (0-0.051) |
|  |  | Kadando | 0.005 (0-0.030) | 0.005 (0-0.047) | 0.007 (0-0.042) | 0.009 (0-0.041) |
|  | West Usambara | |  |  |  |  |
|  |  | Kwadoe | 0.005 (0-0.040) | 0.004 (0-0.019) | 0.005 (0-0.029) | 0.008 (0-0.051) |
|  |  | Funta | 0.005 (0-0.031) | 0.004 (0-0.035) | 0.005 (0-0.065) | 0.005 (0-0.045) |
|  |  | Tamota | 0.005 (0-0.041) | 0.004 (0-0.031) | 0.004 (0-0.025) | 0.006 (0-0.033) |
|  |  | Mgila | 0.006 (0-0.040) | 0.005 (0-0.035) | 0.008 (0-0.053) | 0.007 (0-0.042) |
|  | Tanga coast | |  |  |  |  |
|  |  | Mgome | 0.004 (0-0.030) | 0.004 (0.022) | 0.005 (0-0.026) | 0.006 (0-0.046) |

**Supplementary Table 2**

Background information of the 11 villages where α^3.7^-globin genotyping was not attempted.

| Transect (region), village | | | Altitude, m | Sample size, n | Major ethnic group, % | Females, % | Malaria parasite prevalence, % | Mild anaemia prevalence, % |
| --- | --- | --- | --- | --- | --- | --- | --- | --- |
|  |  |  |  |  |  |  |  |  |
|  | North Pare (Kilimanjaro) | |  |  |  |  |  |  |
|  |  | Kilomeni | 1556 | 322 | Wapare (98.0) | 54.4 | 2.8 | 17.4 |
|  |  | Lambo | 1187 | 275 | Wapare (96.4) | 60.2 | 2.5 | 15.3 |
|  |  | Ngulu | 831 | 386 | Wapare (93.0) | 56.7 | 6.0 | 21.5 |
|  |  | Kambi ya Simba | 745 | 234 | Wapare (79.7) | 51.7 | 10.3 | 22.0 |
|  | West Usambara 1 (Tanga) | |  |  |  |  |  |  |
|  |  | Emmao | 1845 | 190 | Wasambaa (60.8) | 64.0 | 3.7 | 17.4 |
|  |  | Handei | 1425 | 383 | Wasambaa (94.8) | 54.6 | 25.8 | 40.5 |
|  |  | Tewe | 1049 | 347 | Wasambaa (93.9) | 64.5 | 33.4 | 34.1 |
|  |  | Mng’alo | 416 | 373 | Wasambaa (89.5) | 58.1 | 47.8 | 56.3 |
|  | West Usambara 2 (Tanga)^*^ | |  |  |  |  |  |  |
|  |  | Magamba | 1685 | 218 | Wasambaa (69.3) | 55.0 | 3.2 | 17.4 |
|  |  | Ubiri | 1216 | 165 | Wasambaa (98.1) | 62.2 | 16.6 | 29.1 |
|  |  | Kwemasimba | 662 | 242 | Wasambaa (93.7) | 56.2 | 24.3 | 47.1 |

^*^ The coastal village of Mgome (Table 1) is deemed from this transect according to a previous sudy (Drakeley et al, 2005).

**Supplementary Table 3**

Association analysis after carrying out genotype imputation through IM_0_ and IM_1_ using data from: (i) the 13 villages where α^3.7^-globin genotyping was attempted in the majority of the individuals, (ii) the same 13 villages and an additional village where α^3.7^-globin genotyping was not attempted, and (iii) all the 24 villages.

| Analysis | | | IM_0_ | | | |  | IM_1_ | | | |
| --- | --- | --- | --- | --- | --- | --- | --- | --- | --- | --- | --- |
|  |  |  | Mean association signal (range) ^a^ | Estimates (SE) | | |  | Mean association signal (range) ^a^ | Estimates (SE) | | |
|  |  |  |  | Mean genotype | λ_1_ | λ_2_ |  |  | Mean genotype | λ_1_ | λ_2_ |
| 13 villages^b^ | | | 0.74 (0.01-2.47) | 0.334 (0.010) | -0.139 (0.125) | 0.170 (0.270) |  | 1.38 (0.18-3.69) | 0.326 (0.010) | -0.223 (0.133) | 0.132 (0.272) |
| 13 villages and an additional village^c^ | | |  |  |  |  |  |  |  |  |  |
|  | North Pare | |  |  |  |  |  |  |  |  |  |
|  |  | Kilomeni | 0.70 (0.00-2.74) | 0.333 (0.009) | -0.115 (0.129) | 0.193 (0.269) |  | 1.51 (0.21-3.63) | 0.328 (0.013) | -0.234 (0.129) | 0.130 (0.276) |
|  |  | Lambo | 0.72 (0.06-2.14) | 0.335 (0.010) | -0.119 (0.120) | 0.219 (0.264) |  | 1.56 (0.17-4.39) | 0.325 (0.014) | -0.236 (0.136) | 0.150 (0.268) |
|  |  | Ngulu | 0.77 (0.05-2.18) | 0.335 (0.010) | -0.132 (0.125) | 0.190 (0.265) |  | 1.52 (0.38-3.35) | 0.328 (0.015) | -0.235 (0.123) | 0.144 (0.261) |
|  |  | Kambi ya Simba | 0.74 (0.03-2.30) | 0.334 (0.010) | -0.130 (0.125) | 0.182 (0.259) |  | 1.46 (0.15-3.16) | 0.327 (0.013) | -0.229 (0.125) | 0.144 (0.260) |
|  | West Usambara 1 | |  |  |  |  |  |  |  |  |  |
|  |  | Emmao | 0.69 (0.01-2.19) | 0.334 (0.010) | -0.117 (0.121) | 0.204 (0.270) |  | 1.41 (0.17-3.42) | 0.327 (0.012) | -0.232 (0.124) | 0.109 (0.271) |
|  |  | Handei | 0.70 (0.03-2.04) | 0.334 (0.009) | -0.115 (0.115) | 0.190 (0.256) |  | 1.61 (0.07-5.13) | 0.328 (0.015) | -0.221 (0.129) | 0.158 (0.269) |
|  |  | Tewe | 0.74 (0.03-1.97) | 0.335 (0.009) | -0.114 (0.122) | 0.190 (0.254) |  | 1.57 (0.12-4.07) | 0.327 (0.013) | -0.227 (0.125) | 0.135 (0.255) |
|  |  | Mn’galo | 0.55 (0.13-1.61) | 0.335 (0.010) | -0.093 (0.116) | 0.172 (0.248) |  | 1.78 (0.14-4.29) | 0.328 (0.014) | -0.240 (0.125) | 0.127 (0.270) |
|  | West Usambara 2 | |  |  |  |  |  |  |  |  |  |
|  |  | Magamba | 0.72 (0.01-2.76) | 0.334 (0.010) | -0.111 (0.130) | 0.216 (0.268) |  | 1.47 (0.12-3.70) | 0.325 (0.010) | -0.235 (0.125) | 0.125 (0.272) |
|  |  | Ubiri | 0.66 (0.04-2.08) | 0.335 (0.010) | -0.116 (0.122) | 0.183 (0.263) |  | 1.38 (0.18-3.37) | 0.329 (0.009) | -0.223 (0.128) | 0.109 (0.268) |
|  |  | Kwemasimba | 0.76 (0.06-2.46) | 0.336 (0.010) | -0.109 (0.122) | 0.226 (0.257) |  | 1.54 (0.34-4.13) | 0.338 (0.010) | -0.226 (0.121) | 0.117 (0.269) |
| 24 villages^c^ | | | 0.53 (0.01-2.29) | 0.335 (0.008) | -0.047 (0.097) | 0.170 (0.227) |  | 2.67 (0.31-8.82) | 0.340 (0.034) | -0.238 (0.117) | 0.140 (0.254) |

^a^ Association signal is calculated by –log_10_(p-value) using either the mean or the median of log-likelihood ratio statistic across all imputed data sets.

^b^ Results based on 100 imputed datasets generated by MICE using chains of 25 iterations and random initial conditions.

^c^ Results based on 100 imputed datasets generated by MICE using chains of 100 iterations and random initial conditions.
